# Intelligent maneuver decision-making for UAVs using the TD3–LSTM reinforcement learning algorithm under uncertain information

**DOI:** 10.3389/frobt.2025.1645927

**Published:** 2025-08-01

**Authors:** Tongle Zhou, Ziyi Liu, Wenxiao Jin, Zengliang Han

**Affiliations:** College of Automation Engineering, Nanjing University of Aeronautics and Astronautics, Nanjing, China

**Keywords:** unmanned aerial vehicles, maneuver decision-making, reinforcement learning, twin delayed deep deterministic policy gradient, long short-time memory

## Abstract

Aiming to address the complexity and uncertainty of unmanned aerial vehicle (UAV) aerial confrontation, a twin delayed deep deterministic policy gradient (TD3)–long short-term memory (LSTM) reinforcement learning-based intelligent maneuver decision-making method is developed in this paper. A victory/defeat adjudication model is established, considering the operational capability of UAVs based on an aerial confrontation scenario and the 3-degree-of-freedom (3-DOF) UAV model. For the purpose of assisting UAVs in making maneuvering decisions in continuous action space, a model-driven state transition update mechanism is designed. The uncertainty is represented using the Wasserstein distance and memory nominal distribution methods to estimate the detection noise of the target. On the basis of TD3, an LSTM network is utilized to extract features from high-dimensional aerial confrontation situations with uncertainty. The effectiveness of the proposed method is verified by conducting four different aerial confrontation simulation experiments.

## 1 Introduction

Unmanned aerial vehicles (UAVs) have undergone significant development over recent years, offering advantages such as cost-effectiveness, strong maneuverability, good stealth performance, and the ability to be recycled and reused. It will gradually replace manned aircraft in future complex environments for performing regional reconnaissance, attacking targets, and other tasks (Shin et al., 2018; Zhou et al., 2020b). The process by which UAVs automatically make control decisions by simulating pilots and commanders who respond to various air combat situations is a crucial component of the autonomous decision-making system for aerial confrontations (Zhou et al., 2020a; Wang et al., 2020). As a result, it is critical to develop an intelligent maneuver decision-making approach to enhance UAV autonomy, intelligence, and air combat capability and adapt to the real-time demands of unmanned aerial confrontation.

The OODA (observation, orientation, decision, and action) loop theory governs maneuver decision-making in UAV confrontations (Virtanen et al., 2006). The UAV maneuver decision-making theory has gained significant attention and has been widely studied over the past decade due to advancements in the autonomy and intelligence of UAVs. At the moment, research on the intelligent maneuver decision-making method of UAVs is centered on three areas: expert systems, game theory, and learning algorithms.

Expert system-based maneuver decision-making formulates predicate logic production rules following “if–else–then” principles, upon which UAVs base tactical maneuver selections (Virtanen et al., 2006). The expert system is widely used in actual maneuver decision-making systems due to its simplicity in design and the interpretability of the decision outcomes. However, the expert system is overly reliant on rule dependability and lacks scalability. To improve the adaptability of expert systems in aerial combat, Liu et al. (2016) developed a receding horizon control-based maneuver decision-making method. Tan et al. (2022) developed a fuzzy expert system for UAV short-range escape maneuvers by learning tactical information.

Based on optimization theory, aerial confrontation is considered a strategic game involving decision-makers. The state transition during this process is described by a differential equation (payment function), and the maneuver decision-making problem is subsequently resolved through numerical optimization techniques (Park et al., 2016; Duan et al., 2015). Li Q. et al. (2022) established a decision-making model for maneuver games. It was based on positional situation information, fighter performance, intentional threat, and the collaborative effects of multiple fighters. The optimal decision scheme for the game was determined using the double game tree distributed Monte Carlo search strategy. Duan et al. (2023) designed a game with a mixed objective function for UAV autonomous maneuver decision-making problems, and the optimal solution was obtained using improved pigeon-inspired optimization.

With the accelerated advancement of artificial intelligence technology and computer processing power, deep learning and reinforcement learning algorithms have grown in popularity and are widely employed in unmanned systems. For the deep learning-based maneuver decision-making method, the situation information and UAV performance parameters are input into the deep networks, and the maneuvering action or control command is obtained after training and learning (Zhang and Huang, 2020). Relatively, the idea of reinforcement learning is more aligned with maneuver decision-making of UAVs. Based on the reinforcement learning Markov decision process, a UAV can select the corresponding maneuvering action through the real-time assessment of the environmental situation (Zhang et al., 2018; Tu et al., 2021). A heuristic deep deterministic policy gradient (DDPG) algorithm was introduced to improve the exploration capability in continuous action space for the UAV maneuver decision-making problem (Wang et al., 2022). A deep reinforcement learning and Monte Carlo tree search-based maneuver decision-making method, independent of human knowledge, was proposed by Zhang et al. (2022). Utilizing self-play, the system initiates with random actions to generate air combat training samples (including states, actions, and rewards).

Due to the complexity of the environment in actual aerial confrontations, the traditional discrete maneuver action library struggles to meet the demand for maneuvering diversity. The learning and training of continuous maneuvers require higher algorithm real-time efficiency. The twin delayed deep deterministic policy gradient algorithm (TD3) is a deterministic strategy reinforcement learning algorithm designed for high-dimensional continuous action spaces. It offers significant advantages in offline training plasticity and the real-time usage of neural networks (Hong et al., 2021). Furthermore, the long short-term memory (LSTM) network can transform the aerial confrontation state with uncertainty into a high-dimensional perceptual situation and improve the neural network learning ability of the target state time series data (Kant et al., 2023). Hence, the TD3–LSTM reinforcement learning-based intelligent algorithm is developed to address the UAV maneuver decision-making problem under uncertain information. The following are the major contributions:

• A victory/defeat adjudication model is established based on the actual UAV aerial confrontation scenario, which could ensure the validity of maneuver decision-making.

• A model-driven state transition update mechanism is developed based on the 3-degree-of-freedom (3-DOF) UAV model to ensure the efficiency of the continuous action space.

• A Wasserstein distance-based model aims to describe uncertainty in confrontation, which can enhance the robustness of maneuver decision-making.

• A reinforcement learning intelligent algorithm is proposed based on TD3–LSTM to improve the efficiency of maneuver decision-making.

The remainder of this paper is organized as follows. Section 2 details the problem formulation, including the one-to-one confrontation model, the victory/defeat adjudication model, and the maneuver decision-making system structure. The deep reinforcement learning-based UAV maneuver decision-making method, which consists of a model-driven state transition update mechanism, reward function design, uncertainty description, and the TD3–LSTM algorithm, is introduced in Section 3. In Section 4, we provide the simulation results. Finally, the conclusion is presented in Section 5.

## 2 Problem description

Maneuver decision-making is the process by which a UAV selects maneuvering actions based on the current aerial confrontation situation and environmental information, aiming to gain operational superiority by altering the aerial confrontation dynamics. The maneuver decision problem belongs to the top-level decision problem of UAVs.

### 2.1 Confrontation model of UAV

**FIGURE 1:**
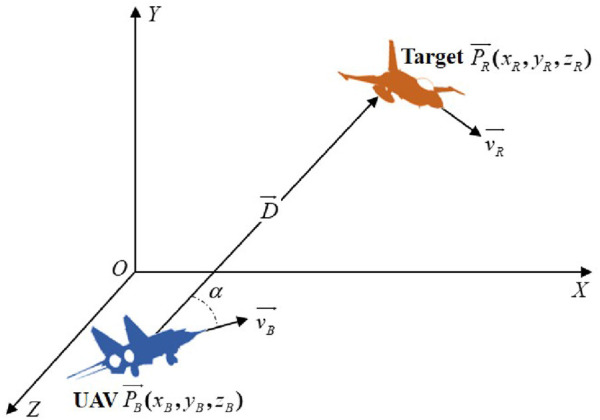
UAV one-on-one confrontation scenario.

The UAV one-on-one confrontation scenario is shown in Figure 1 (Yang et al., 2019).

In Figure 1, $\vec{v_{B}}$ and $\vec{P_{B}}\left(x_{B},y_{B},z_{B}\right)$ denote the velocity vector and position vector of the UAV, respectively, while $\vec{v_{R}}$ and $\vec{P_{R}}\left(x_{R},y_{R},z_{R}\right)$ represent the velocity vector and position vector of the target, respectively. α represents the relative azimuth angle.

Defining $\vec{D}$ as the relative distance of the UAV with respect to the target—where the direction represents the UAV pointing toward the target and the magnitude is given by d — $\vec{D}$ and d can be calculated by Equations 1, 2 (Li B. et al., 2022)

$$\vec{D}=\vec{D_{R}}-\vec{D_{B}},$$ (1)

$$d={\left\|\vec{D}\right\|}_{2}.$$ (2)

Thus, the relative azimuth angle α can be calculated as Equation 3 (Li B. et al., 2022):

$$\alpha =\arccos\left(\left(\vec{D}\times \vec{v_{B}}\right)/\left({\left\|\vec{D}\right\|}_{2}\cdot {\left\|\vec{v_{B}}\right\|}_{2}\right)\right).$$ (3)

### 2.2 Victory/defeat adjudication model

**FIGURE 2:**
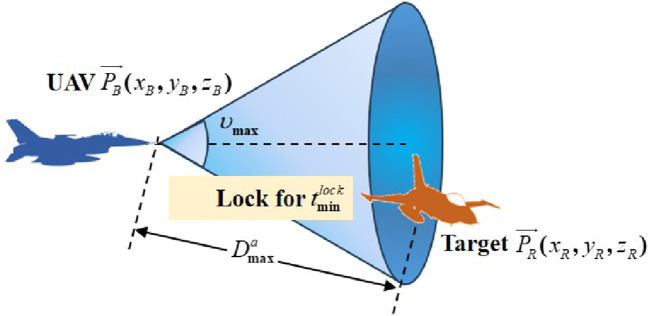
Schematic of the UAV attacking and locking the target.

Generally, the operational capability of a UAV is constrained by the capabilities of its weapon system (Luo et al., 2022). A schematic showing the UAV attacking and locking onto the target is presented in Figure 2.

In Figure 2, $\upsilon_{\max}$ denotes the maximum off-axis emission angle of the UAV weapon system, $D_{\max}^{a}$ stands for the maximum firing distance, and $t_{\min}^{\mathrm{lock}}$ indicates the minimum locking time. Moreover, the UAV wins the confrontation when it locks onto the target for more than $t_{\min}^{\mathrm{lock}}$ seconds within the UAV firing range. Specifically, the victory adjudication condition can be described as Equation 4:

$$\left\{\begin{array}{c} d\leq D_{\max}^{a}\;\\ \alpha \leq \upsilon_{\max}\;\\ t_{in}\geq t_{\min}^{\mathrm{lock}}\; \end{array}\right.,$$ (4)

where $t_{in}$ represents the time the UAV locks onto the target.

Based on the points discussed above, the objective of this paper is to design an intelligent algorithm that enables UAVs to make autonomous decisions to achieve victory adjudication conditions in advance, based on environmental and situational information.

### 2.3 Maneuver decision-making system structure of UAVs

**FIGURE 3:**
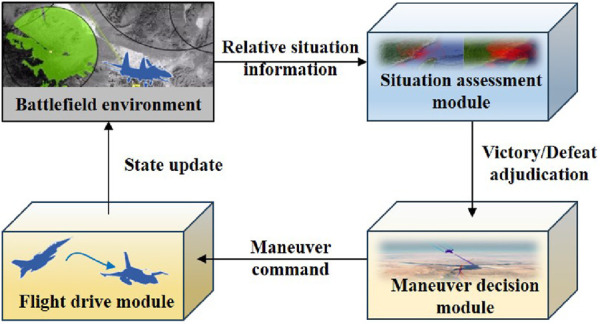
Interactive procedure of the UAV maneuver decision-making system.

The UAV maneuver decision-making system is comprised of three components: the situation assessment module, the maneuver decision-making module, and the flight drive module. The situation assessment module obtains relative situational information from the environment and determines whether the conditions for adjudicating victory are met. If not, the maneuver decision module provides the maneuver command based on the situation assessment result. Afterward, the flight drive module updates the UAV state and provides feedback to the environment. This cycle would continue until one side achieves victory through adjudication. The interactive procedure is shown in Figure 3.

## 3 Deep reinforcement learning-based UAV maneuver decision-making algorithm

**FIGURE 4:**
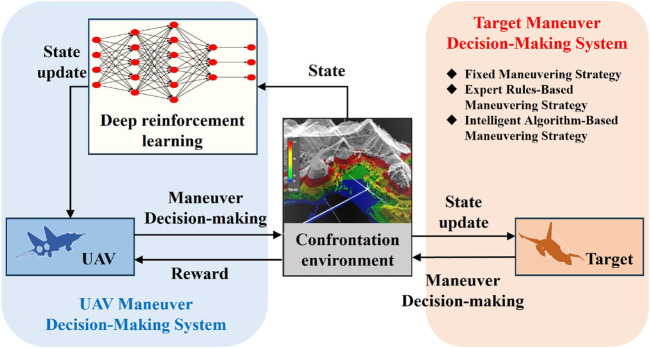
Basic framework of the deep reinforcement learning-based UAV maneuver decision-making algorithm.

To ensure that UAVs meet the victory adjudication condition ahead of schedule, a deep reinforcement learning-based algorithm is proposed in this paper for UAV maneuver decision-making. The basic framework is shown in Figure 4.

### 3.1 UAV model-driven state transition update mechanism

**FIGURE 5:**
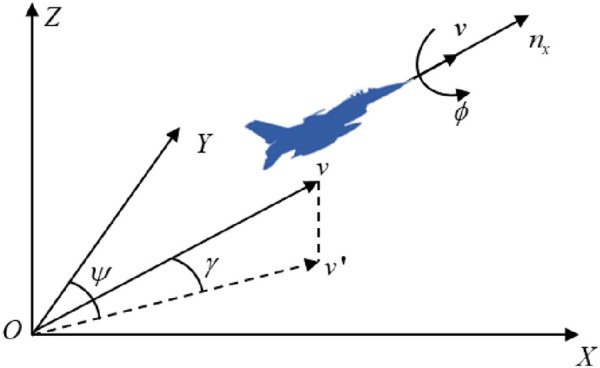
Schematic diagram of the UAV motion model.

The maneuver action of the UAV is designed as a discrete set by NASA, which only considers several basic maneuvers, including uniform flight, accelerated flight, decelerated flight, left turn, right turn, forward climb, and forward dive (Huang et al., 2018). To further describe the maneuvering behavior of the UAV with continuous state in actual aerial confrontations, this paper establishes a state transition update mechanism based on the UAV motion model. The schematic diagram of the UAV motion model is shown in Figure 5 (Guo et al., 2023).

In Figure 5, v , ψ , γ , and ϕ represent the velocity, yaw angle, flight path angle, and roll angle, respectively.

The following particle motion model (Guo et al., 2023) of UAVs is considered in this paper.

$$\left\{\begin{array}{c} \dot{x}=v\cos\gamma \sin\psi \;\\ \dot{y}=v\cos\gamma \cos\psi \;\\ \dot{z}=v\sin\gamma \;\\ \dot{v}=g\left(n_{x}-\sin\gamma \right)\;\\ \dot{\psi }=-\frac{gn_{z}\sin\phi }{v\cos\gamma }\;\\ \dot{\gamma }=\frac{g}{v}\left(n_{z}\cos\phi -\cos\gamma \right)\; \end{array}\right.,$$ (5)

where x , y , and z denote the position of the UAV in the inertial coordinate system. $\dot{x}$ , $\dot{y}$ , and $\dot{z}$ denote the projections of velocity v in the x -axis, y -axis, and z -axis, respectively. $n_{x}$ and $n_{z}$ denote the normal overload and tangential overload of the UAV, respectively. g is the acceleration of gravity.

**FIGURE 6:**
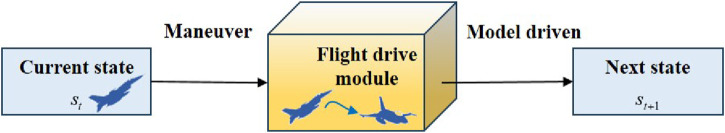
Schematic diagram of UAV dynamic model.

According to Equation 5, the state of the UAV is affected by normal overload $n_{x}$ , tangential overload $n_{z}$ , and roll angle ϕ . Thus, the 3-DOF model mentioned above is established as a flight drive module in this paper. The new state of the UAV is calculated in real-time based on the current state and control input. The UAV motion model-driven state transition update mechanism is shown in Figure 6.

### 3.2 Design of the reward function

As common knowledge dictates, there are four possible scenarios in an aerial confrontation, depending on the relative positions of the UAV and the target: the trailing side holds the advantage, the pursued side is at a disadvantage, and an equilibrium state is reached when both sides are flying either toward or away from each other. To enable UAVs to reach positions with more favorable environmental conditions, this paper considers the instantaneous aerial situation between the UAV and the target, along with the victory/defeat adjudication model, as the basis for reward and punishment signals.

The angle reward $r_{\alpha }$ is defined as Equation 6 (He et al., 2020):

$$r_{\alpha }=\left\{\begin{array}{c} 1-\frac{\alpha_{B}+\alpha_{R}}{2\pi },d\leq D_{\max}^{a}\;\\ \left(1-\frac{\alpha_{B}+\alpha_{R}}{2\pi }\right)e^{-\frac{{\left(d-D_{\max}^{a}\right)}^{2}}{{2D_{\max}^{a}}^{2}}},d>D_{\max}^{a}\; \end{array}\right.,$$ (6)

where $\alpha_{B}$ and $\alpha_{R}$ represent the relative azimuth angle of the UAV and the target, respectively.

When the target is within the attack range of the UAV, the distance reward of the UAV to the target $r_{B\to R}$ is defined as Equation 7 (He et al., 2020):

$$r_{B\to R}=\left\{\begin{array}{c} 5,d\leq D_{\max}^{a}\;and\;\alpha_{B}<\upsilon_{\max}\;and\;\alpha_{R}<\pi /2\;\\ 0,else\; \end{array}\right..$$ (7)

Similarly, when the UAV is within the attack range of the target, the distance reward of the target to the UAV $r_{R\to B}$ is defined as follows (He et al., 2020):

$$r_{R\to B}=\left\{\begin{array}{c} 5,d\leq D_{\max}^{a}\;and\;\alpha_{R}<\upsilon_{\max}\;and\;\alpha_{B}<\pi /2\;\\ 0,else\; \end{array}\right..$$ (8)

Invoking Equations 8, 9, the distance reward $r_{d}$ is defined as follows (He et al., 2020):

$$r_{d}=r_{B\to R}-r_{R\to B}.$$ (9)

In addition, to ensure the UAV flight safety and avoid collisions, the height reward $r_{h}$ is defined as Equation 10 (Li et al., 2021):

$$r_{h}=\left\{\begin{array}{cc} -10,\; & 1km<h<12km\;and\;d>200m\\ 0,\; & else \end{array}\right.,$$ (10)

where h represents the flight height of the UAV.

In summary, the total reward R is obtained by Equation 11:

$$R=r_{\alpha }+r_{d}+r_{h}.$$ (11)

### 3.3 Uncertain information of maneuver decision-making

Due to the uncertainties in the UAV model and the complexity and flexibility of the actual aerial confrontation environment, the UAV and the target may not be able to reach the desired position after maneuvering during the aerial confrontation.

To describe the uncertainty of aerial confrontation, we assume that the state deviation Δs after a maneuver action follows a discrete empirical distribution $\hat{\Omega }$ constructed from observations. The nominal distribution $\hat{\Omega }$ can be indirectly observed through the historical data samples $O_{Hi}$ , where i∈{1,2,…,N} , and the current data samples $O_{Cj}$ , where j∈{1,2,…,M} .

Under the uniform distribution over the N historical data samples and M current data samples, we have

$$\hat{\Omega }=\frac{1}{N+\lambda M}\overset{N}{\underset{i=1}{\sum }}\xi_{O_{Hi}}+\frac{\lambda }{N+\lambda M}\overset{N}{\underset{i=1}{\sum }}\xi_{O_{Cj}},$$ (12)

In Equation 12, $\xi_{O_{Hi}}$ denotes the Dirac point mass at the i th historical data sample $O_{Hi}$ , $\xi_{O_{Cj}}$ denotes the Dirac point mass at the j th current data sample $O_{Cj}$ , and λ>1 is the proportional parameter, representing that the current data samples are more valuable.

Moreover, to describe the true distribution of target state deviation Δs , the Wasserstein distance is considered to measure the distance between any two distributions. Lemma 1 defines the distance.

Lemma 1 (Wasserstein distance (Esfahani and Kuhn, 2018)): The Wasserstein distance between any probability distributions Θ and the nominal distribution $\hat{\Omega }$ is defined as Equation 13:

$$W_{p}\left(\Theta ,\hat{\Omega }\right)=\underset{\pi \in \Pi \left(\Theta ,\hat{\Omega }\right)}{\inf}\int_{\Xi \times \Xi }\left\|\Delta s-\Delta s^{\prime }\right\|\pi \left(d\Delta s,d\Delta s^{\prime }\right),$$ (13)

where $\left\|\cdot \right\|$ is a norm and $\Pi \left(\Theta ,\hat{\Omega }\right)$ is the set of all joint probability distributions of Δs and $\Delta s^{\prime }$ with the marginals Θ and $\hat{\Omega }$ , respectively.

According to Lemma 1, the true distribution of the state deviation Δs is defined in a set as Equation 14:

$$\Lambda \left(\hat{\Omega }\right)=\left\{\Delta s\in P\left(\Xi \right):W_{p}\left(\Theta ,\hat{\Omega }\right)\leq \eta_{k}\right\},$$ (14)

where Ξ is a closed set containing all possible values of Δs , P(Ξ) is the family of all probability distributions supported on Ξ , and $\eta_{k}$ is the maximum Wasserstein distance.

**FIGURE 7:**
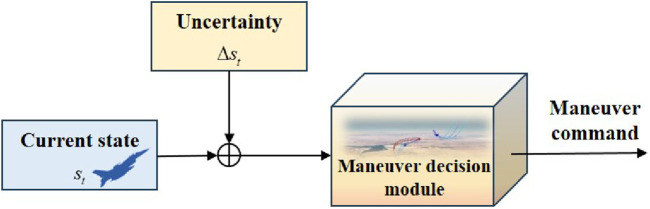
Schematic diagram of maneuver decision-making uncertainty.

On the basis of this, the state deviation Δs is considered to ensure accuracy in this paper. The schematic diagram is shown in Figure 7.

### 3.4 TD3–LSTM algorithm

The TD3 algorithm is a novel form of deep reinforcement learning algorithm, founded on the policy gradient algorithm and the actor–critic (AC) framework (Cheng et al., 2021; Duan et al., 2022). The proposed UAV model-driven state transition update mechanism in this paper is suitable and can be applied in continuous state and action spaces.

The TD3 algorithm consists of six networks, namely, the actor network $\pi_{\mu }$ , the critic 1 network $Q_{\theta_{1}}$ , the critic 2 network $Q_{\theta_{2}}$ , the target actor network $\pi_{\mu^{\prime }}$ , the target critic 1 network $Q_{\theta_{1}^{\prime }}$ , and the target critic 2 network $Q_{\theta_{2}^{\prime }}$ . The network parameters for each network are μ , $\theta_{1}$ , $\theta_{2}$ , $\mu^{\prime }$ , $\theta_{1}^{\prime }$ , and $\theta_{2}^{\prime }$ . Based on the two critic networks’ structure, the TD3 algorithm selects the smaller Q value to alleviate overestimation.

In each episode, TD3 selects an action $a∼\pi_{\phi }\left(s\right)+\epsilon ,\epsilon ∼N\left(0,\sigma \right)$ , using exploration noise, and observes reward r and new state $s^{\prime }$ , where ϵ denotes the noise added to the output of the policy network to enhance the stability of the algorithm, as specified in Equation 15 (Fujimoto et al., 2018).

$$\tilde{a}\leftarrow \pi_{\varphi^{\prime }}\left(s^{\prime }\right)+\varepsilon ,\varepsilon ∼\mathrm{clip}\left(N\left(0,\tilde{\delta }\right),-c,c\right).$$ (15)

Based on the structure of the two critic networks, the TD3 algorithm selects the minimum between the two estimates of $Q_{\theta_{1}}$ and $Q_{\theta_{2}}$ to avoid overestimation. The objective function of the TD3 algorithm is defined as Equation 16 (Fujimoto et al., 2018):

$$y\leftarrow r+\gamma \underset{i=1,2}{\min}Q_{\theta_{i}^{\prime }}\left(s^{\prime },\tilde{a}\right).$$ (16)

The TD-error $e_{TD}$ of $Q_{\theta_{1}}$ and $Q_{\theta_{2}}$ is defined as Equation 17 (Fujimoto et al., 2018):

$$e_{TD}=y-Q_{\theta_{i}}\left(s,a|\theta_{i}\right).$$ (17)

The critics can be updated as Equation 18 (Fujimoto et al., 2018):

$$\theta_{i}\leftarrow {\mathrm{argmin}}_{\theta_{i}}N^{-1}\sum {\left(y-Q_{\theta_{i}}\left(s,a\right)\right)}^{2}.$$ (18)

The actor network is updated via the deterministic policy gradient as Equation 19 (Fujimoto et al., 2018):

$$\nabla_{\mu }J\left(\mu \right)=\left.{\left.N^{-1}\sum \nabla_{a}Q_{\theta_{1}}\left(s,a\right)\right|}_{a=\pi_{\mu }\left(s\right)}\nabla_{\mu }\pi \right|\mu \left(s\right).$$ (19)

The target networks are updated through a slow-moving update rate τ , following Equations 20, 21. Specifically,

$$\theta_{i}^{\prime }\leftarrow \tau \theta_{i}+\left(1-\tau \right)\theta_{i}^{\prime },$$ (20)

$$\mu^{\prime }\leftarrow \tau \mu +\left(1-\tau \right)\mu^{\prime }.$$ (21)

**FIGURE 8:**
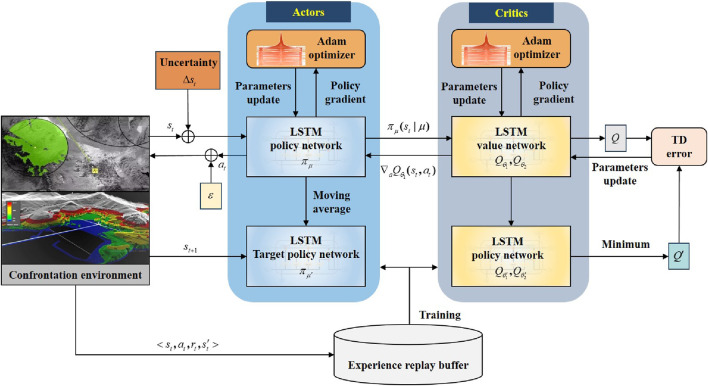
Architecture diagram of the TD3–LSTM algorithm.

Due to the high dynamic and high-dimensional complexity in an actual aerial confrontation environment, the TD3 algorithm cannot effectively manage uncertainty and model the policy function and value function using a fully connected neural network. By adding a special gate structure to RNN, the LSTM network has a positive impact on processing time series data, thus enhancing the efficiency and effectiveness of the training algorithm (T. Ergen, 2018). It is considered that the state information of UAVs and targets in actual aerial confrontations exhibits time series characteristics. In this paper, an LSTM network is utilized to extract features from high-dimensional aerial confrontation situations with uncertainty. This structure aims to output valuable perceptual information and advance representation learning for sequential sample data. The policy and value functions are jointly approximated using a fully connected neural network. The TD3–LSTM algorithm architecture diagram is shown in Figure 8.

**FIGURE 9:**
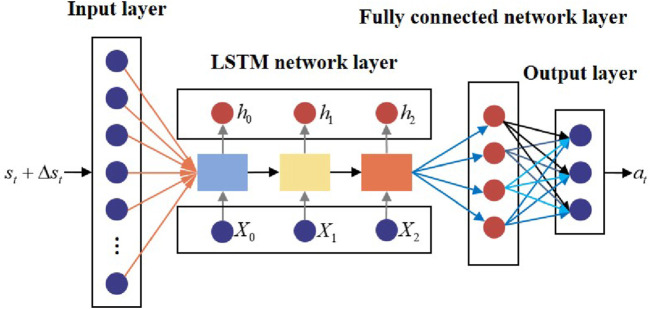
General structure of the LSTM policy network.

The general structure of the LSTM policy network is shown in Figure 9.

The structural parameters of the LSTM policy network are provided in Table 1.

**TABLE 1:** Structural parameters of the LSTM policy network.

Structural parameter	Description
Input layer	Twelve states with uncertainty of the UAV and target
Hidden layer 1	Three LSTM network units
Hidden layer 2	One fully connected network layer
Output layer	Three nodes that correspond to $n_{x}$ , $n_{z}$ , and ϕ of the UAV
Activation function	Hidden layer: ReLU ; output layer: tanh
Training method	Adam

**FIGURE 10:**
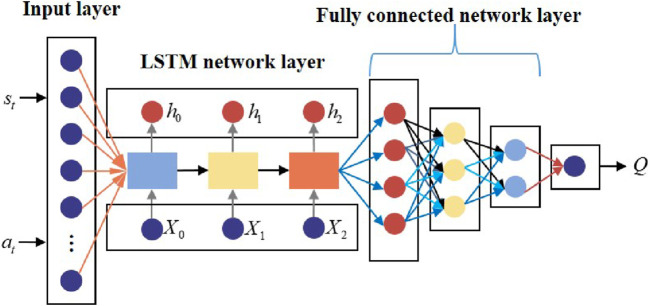
General structure of the LSTM value network.

The general structure of the LSTM value network is presented in Figure 10.

The structural parameters of the LSTM value network are shown in Table 2.

**TABLE 2:** Structural parameters of the LSTM value network.

Structural parameter	Description
Input layer	Fifteen dimensions (current states and action)
Hidden layer 1	Three LSTM network units
Hidden layer 2	Three fully connected network layers
Output layer	One node that corresponds to the Q value
Activation function	Hidden layer: Sigmoid ; output layer: tanh
Training method	Adam

## 4 Simulation

To demonstrate the advantages of the proposed maneuver decision-making method, the simulation is verified in four different scenarios.

### 4.1 Scenario 1: target in straight flight

**FIGURE 11:**
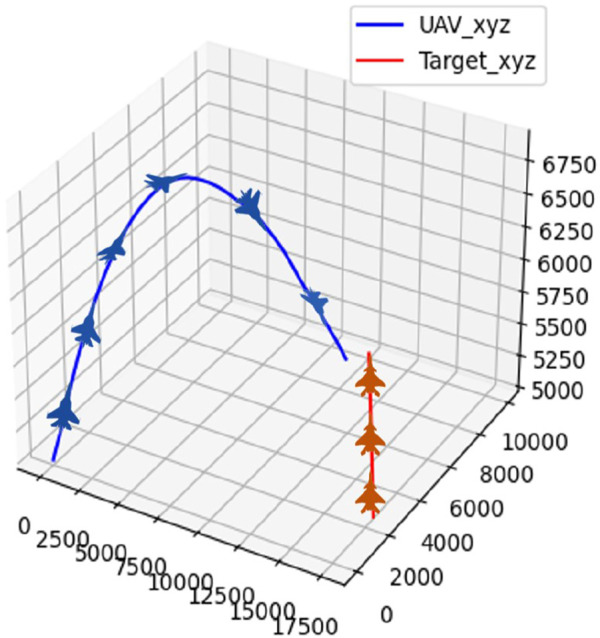
Confrontation trajectory in scenario 1.

The initial position and attitude information of the UAV and the target are randomly initialized, and the target follows a strategy of uniform-speed straight flight. The confrontation trajectory of the UAV and the target in scenario 1 is depicted in Figure 11.

**FIGURE 12:**
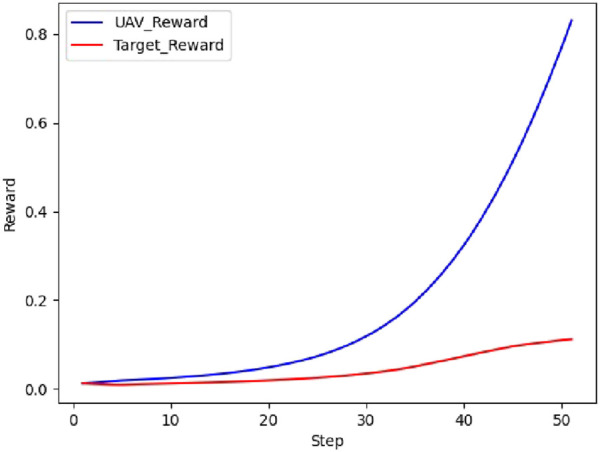
Real-time reward curve in scenario 1.

The real-time reward curve of the UAV and the target in scenario 1 is depicted in Figure 12.

On the basis of Figures 11, 12, the UAV gains a height advantage by climbing and then dives toward the target after reaching a specific altitude to gain velocity and angle advantages. This maneuver forces the target into the attack zone, leading to eventual triumph.

**FIGURE 13:**
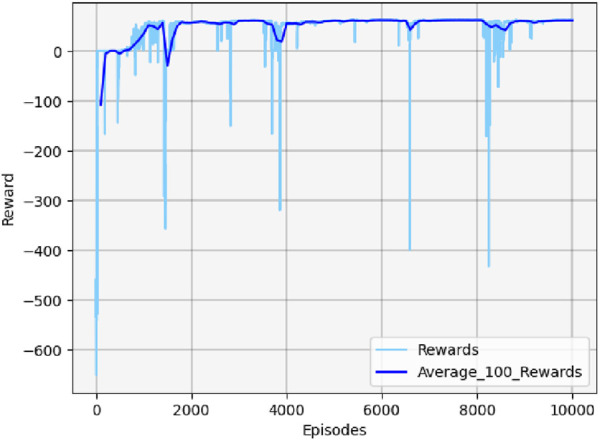
Cumulative reward curve in scenario 1.

Figure 13 shows the cumulative reward curve of the UAV and the target.

In the early stages, the cumulative reward curve fluctuates because the UAV is unable to learn any effective strategies, leading to crashes or defeats in confrontations. With further training, the UAV learned effective maneuvers, developed an attack posture, locked onto the target, and gradually increased its cumulative reward value until convergence. It should be noted that the cumulative reward value may fluctuate slightly during the later stages of training due to uncertainty considerations. However, this variation will not impact the eventual acquisition of effective maneuvering strategies.

### 4.2 Scenario 2: target in circle flight

**FIGURE 14:**
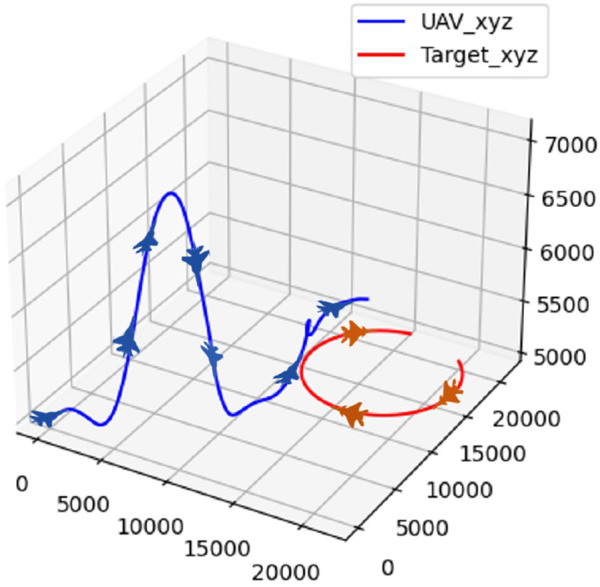
Confrontation trajectory in scenario 2.

In scenario 2, the target employs the circle maneuver strategy. The trajectory of the UAV and the target during the confrontation is illustrated in Figure 14.

**FIGURE 15:**
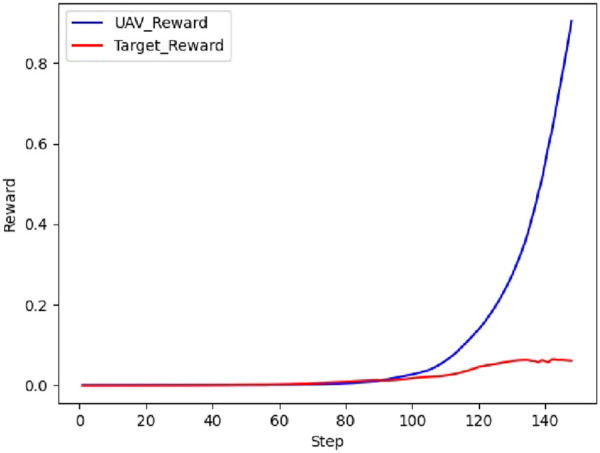
Real-time reward curve in scenario 2.

The real-time reward curve of the UAV and the target in scenario 2 is presented in Figure 15.

According to Figures 14, 15, the UAV climbs to gain a height advantage before diving toward the target to lock onto it for the first time. However, the target continues to circle due to the insufficient lock time. The UAV then ascends to gain a height advantage, locks onto the target again, and maintains the lock until it wins the confrontation.

**FIGURE 16:**
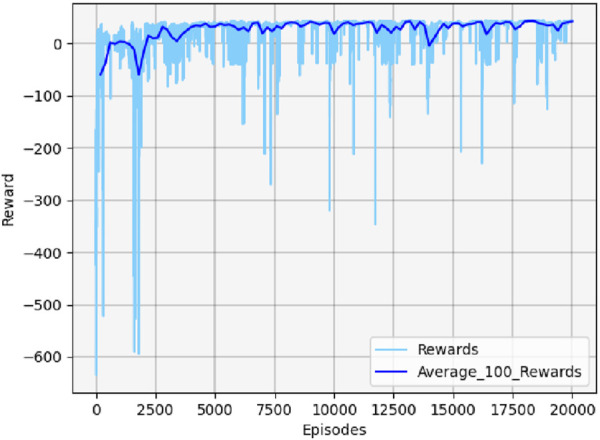
Cumulative reward curve in scenario 2.

Figure 16 illustrates the cumulative reward curve of the UAV and the target in scenario 2.

Similarly, the UAV failed to learn any useful strategies at the beginning of the training. Once the UAV learns efficient maneuvers, the cumulative reward value gradually increases until convergence. The cumulative reward value also fluctuates slightly in the late training period due to the effect of uncertainty.

### 4.3 Scenario 3: expert rule-based target maneuvering strategy

**FIGURE 17:**
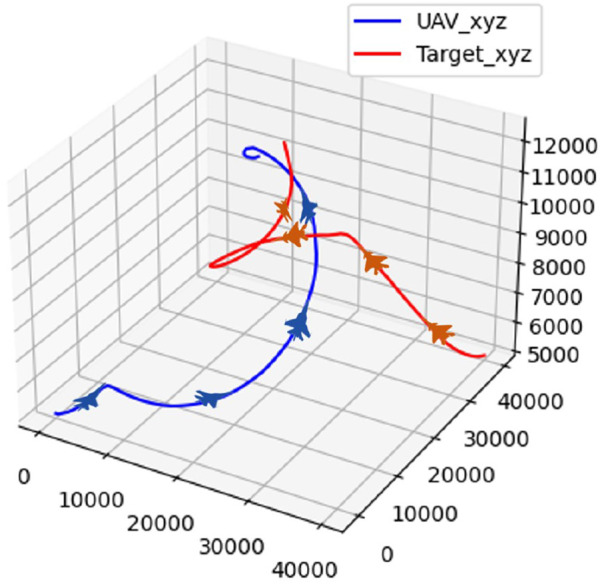
Confrontation trajectory in scenario 3.

In scenario 3, the target selects maneuvers according to expert rules. The confrontation trajectory of the UAV and the target in scenario 3 is illustrated in Figure 17.

**FIGURE 18:**
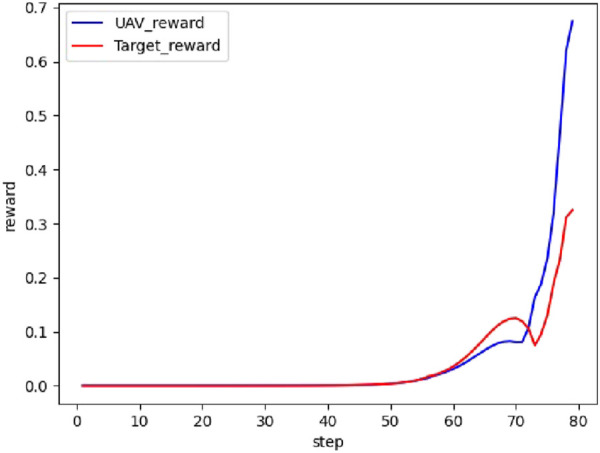
Real-time reward curve in scenario 3.

The real-time reward curve of the UAV and the target in scenario 3 is shown in Figure 18.

From Figures 17, 18, the UAV and the target climb simultaneously to gain a height advantage during the initial phase. Next, both the UAV and the target choose a turning strategy to prevent being locked onto each other. Finally, the UAV performs a somersault maneuver to circle behind the target, completes the lock, and triumphs over the confrontation.

**FIGURE 19:**
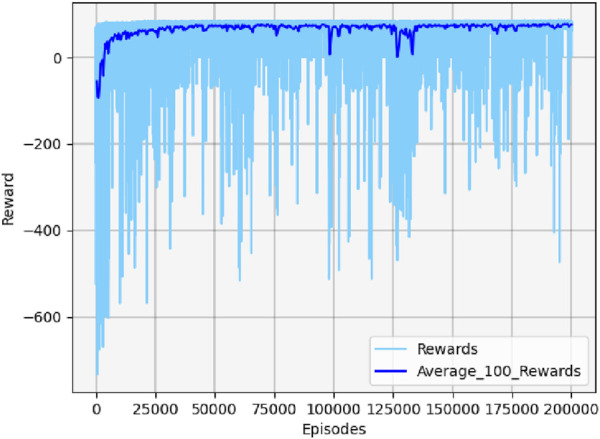
Cumulative reward curve in scenario 3.

Figure 19 shows the cumulative reward curve of the UAV and the target.

Based on the results in Figure 19, the confrontation process is more intensive since the target has a specific maneuvering strategy. Because the UAV lacks environmental cognition, it is unable to develop effective strategies, leading to the UAV acting with high penalty values in the initial stages of training. The trend in the cumulative reward curve change indicates that the convergence speed is relatively slow, and the curve fluctuates sharply, reflecting the difficulty and complexity of aerial confrontation.

### 4.4 Scenario 4: genetic algorithm-based target maneuvering strategy

**FIGURE 20:**
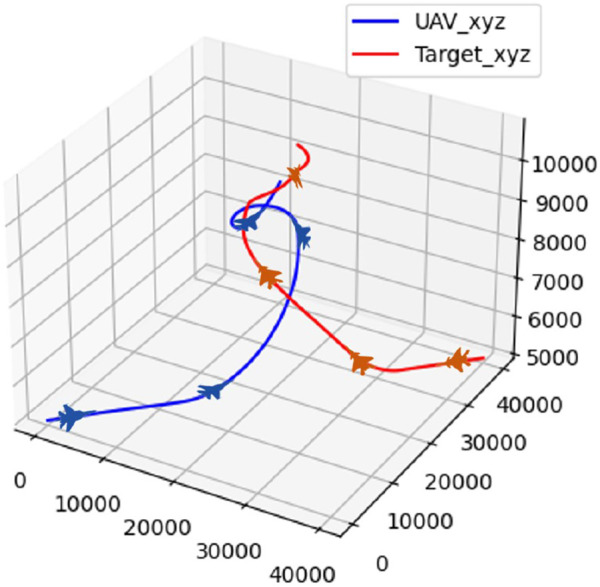
Confrontation trajectory in scenario 4.

In scenario 4, the target selects the optimal maneuvers based on the current situation using a genetic algorithm. The confrontation trajectory of the UAV and the target in scenario 4 is shown in Figure 20.

**FIGURE 21:**
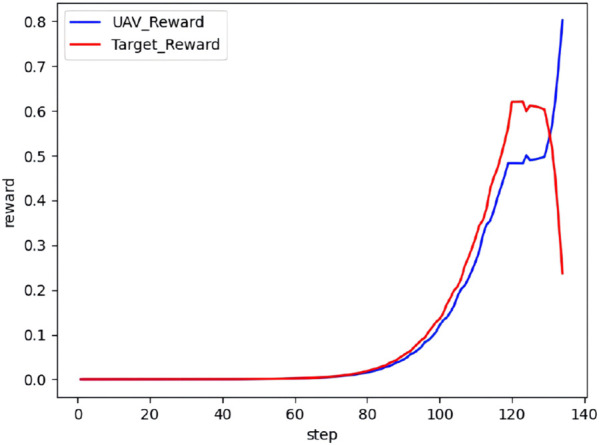
Real-time reward curve in scenario 4.

The real-time reward curve of the UAV and the target in scenario 4 is shown in Figure 21.

As observed from Figures 20, 21, both the UAV and the target climb simultaneously, aiming to gain a height advantage at the start. Next, the target continues to climb in an attempt to gain a vantage point. The UAV takes the opportunity and maneuvers around the rear of the target during this process. Finally, the UAV completes the lock and wins the confrontation.

**FIGURE 22:**
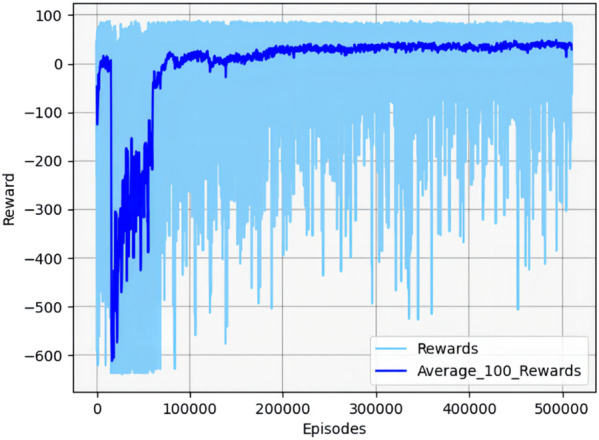
Cumulative reward curve in scenario 4.

Figure 22 shows the cumulative reward curve of the UAV and the target.

As shown in Figure 22, the target selects the optimal maneuver based on the genetic algorithm optimization results under the current situation. The UAV has not yet learned the corresponding strategy and is unable to defeat the target in the early stages of training. The cumulative reward curve fluctuates sharply in the negative area within a certain number of iterations. As the UAV continues to learn the maneuvering strategy, it gradually becomes able to defeat the target. Compared with the three other scenarios, the convergence speed of the cumulative reward curve is slower, and the curve fluctuates sharply in scenario 4.

## 5 Conclusion

In this paper, a TD3–LSTM reinforcement learning-based intelligent algorithm is developed to address the maneuver decision-making problem of a UAV under uncertain information. To ensure the validity, robustness, and efficiency of maneuver decision-making in UAV aerial confrontation scenarios, four simulation experiments are considered in this manuscript: target in straight flight, target in circle flight, target in expert rule-based maneuvering strategy, and genetic algorithm-based strategy. The simulation results demonstrate that regardless of the maneuvering strategy the target adopts, the UAV can comprehend the environmental situation, execute appropriate maneuvering actions, and ultimately emerge victorious in an aerial confrontation. For future work, the implementation of multi-drone collaborative adversarial maneuvering decisions based on higher-fidelity models warrants consideration. Furthermore, achieving efficient sim-to-real policy transfer through transfer learning presents a significant research priority.

## Data Availability

The raw data supporting the conclusions of this article will be made available by the authors, without undue reservation.
